# Potential Anti-Depressive Effects and Mechanisms of Zhi-Zi Hou-Po Decoction Using Behavioral Despair Tests Combined With *in Vitro* Approaches

**DOI:** 10.3389/fphar.2022.918776

**Published:** 2022-07-06

**Authors:** Yongtao Bai, Guoliang Dai, Lihua Song, Xiaolei Gu, Ning Ba, Wenzheng Ju, Wenzhou Zhang

**Affiliations:** ^1^ Department of Pharmacy, The Affiliated Cancer Hospital of Zhengzhou University and Henan Cancer Hospital, Zhengzhou, China; ^2^ Clinical Research Center, The Affiliated Cancer Hospital of Zhengzhou University and Henan Cancer Hospital, Zhengzhou, China; ^3^ Department of Clinical Pharmacology, Affiliated Hospital of Nanjing University of Chinese Medicine, Nanjing, China

**Keywords:** depression, PC12 cells, Zhi-Zi Hou-Po Decoction, mechanisms, BDNF (brain-derived neurotrophic factor)

## Abstract

Zhi-Zi Hou-Po Decoction (ZHD) has been widely used in the treatment of depression for centuries. This study aimed to investigate the antidepressant effects of the water extract of ZHD (ZHD-WE) and ethanol extract of ZHD (ZHD-EE) using behavioral despair tests in mice, and to further explore the neuroprotective effects in a PC12 cell injury model induced by corticosterone (CORT). Mice were divided into a control group (normal saline), ZHD-WE groups (4, 8, and 16 g kg^−1^), ZHD-EE groups (4, 8, and 16 g kg^−1^) and the fluoxetine group (20 mg kg^−1^). The forced swimming test (FST) and tail suspension test (TST) were used to screen the antidepressant effects of ZHD-WE and ZHD-EE after oral administration for seven consecutive days. The level of brain-derived neurotrophic factor (BDNF) in the hippocampus was determined by ELISA. The MTT, lactate dehydrogenase (LDH) and flow cytometry analysis were performed to elucidate the neuroprotective effect of ZHD-EE on a PC12 cell injury model. Additionally, the mRNA and proteins expression of apoptotic molecules Bax, Bcl-2 and BDNF were detected by RT-PCR and western blot assay. It showed that ZHD-EE at concentrations of 8 and 16 g kg^−1^ significantly decreased the immobility time in the TST and FST, and increased the BDNF levels in the hippocampus. While ZHD-WE at concentrations of 4, 8, and 16 g kg^−1^ had no significant effect on the immobility time in the TST, and only the 16 g kg^−1^ of extract group significantly decreased the immobility time in the FST. *In vitro,* the obtained results showed that PC12 cells pre-incubated with ZHD-EE at concentrations of 100 and 400 μg ml^−1^ improved cell viability, decreased LDH release, and reduced apoptosis rate of PC12 cells. Moreover, ZHD-EE significantly increased the mRNA and proteins expression of Bcl-2 and BDNF, while decreased the mRNA and protein expression of Bax. ZHD-EE significantly improved despair-like behavior in mice, and its mechanism may be related to BDNF upregulation in the hippocampus. This study also showed that ZHD-EE had a protective effect on CORT-induced injury in PC12 cells by upregulating the expression of BDNF and restoring Bcl-2/Bax balance.

## Introduction

Depression is a common psychiatric disease that involves genetic, psychological, biochemical, and social factors (Bahramsoltani et al., 2015). According to the World Health Organization, depression disorder will be the main cause of disease burden by 2030 worldwide (Dwyer et al., 2020). Currently, antidepressant drugs based on the monoaminergic system are used as the first-line treatment for depression, which relieves the symptoms of depressed patients to a certain extent. Unfortunately, despite intense research achievements in identifying new drugs for depression, some patients have no response to antidepressant treatments because of the multiple and complicated pathogenic factors involved in depression (Oh et al., 2019). Moreover, some patients have adverse reactions to these drugs, such as fractures, withdrawal symptoms, and antidepressant contraindications (Coupland et al., 2018; Hengartner et al., 2019; Wigmore et al., 2020). Antidepressant drugs are inadequate and far from ideal at present (Zhang and Cheng, 2019). Therefore, it is quite important to look for alternative antidepressant drugs with low toxicity and high efficiency.

Over the past few thousand years, traditional Chinese medicine (TCM), with its advantages of integrated theory for diagnosis and treatment, has been widely used in China, and provides alternative therapies for the treatment of depression (Zhang and Cheng, 2019; Han et al., 2020). Zhi-Zi Hou-Po Decoction (ZHD) is a representative prescription in TCM for the treatment of depression. It was originally described in Treatise on Febrile and Miscellaneous Diseases and composed of *Gardenia jasminoides* J. Ellis, *Magnolia officinalis* Rehd. et Wils., and *Citrus × aurantium* L. In previous study, we have proved the antidepressant effect of ZHD-EE in the animal depressive model induced by corticosterone by regulating the cAMP signaling pathway and monoaminergic metabolism (Bai et al., 2021). There is no comparative study on the antidepressant effects of ZHD-WE and ZHD-EE. This study aimed to investigate the antidepressant effect of the ZHD-WE and ZHD-EE using behavioral despair tests in mice, and to further explore the neuroprotective effect in a PC12 cell injury model induced by CORT.

The activation of hypothalamic-pituitary-adrenal axis is the most consistent biological psychopathology in major depressive disorder (Pariante and Lightman, 2008; Keller et al., 2017). Elevated glucocorticoid concentration is present in the saliva, plasma and urine of some cases with depression, and glucocorticoid levels can be effectively decreased after antidepressant treatment (Parker et al., 2003; Mason and Pariante, 2006; Cai et al., 2020). In addition, studies on rodents have indicated that animals showed obvious depressive symptoms when exposed to stimuli for a long time as a result of elevated levels of corticosterone in the body (Cai et al., 2015; Demuyser et al., 2016). PC12 cells, which have high levels of glucocorticoid receptor expression, possess neuronal-like features and are derived from the rat pheochromocytoma. Thus, the PC12 cell injury model subjected to corticosterone has been widely used in the field of antidepressant drug research for elucidating the neurobiology and underlying mechanism of antidepressants. Gong et al. proved that senkyunolide A protected PC12 cell apoptosis induced by corticosterone by modulating activities of PP2A and α-syn (Gong et al., 2018). Kogilavani et al. had shown that Malaysian *Padina australis* significantly protects PC12 cells damage against CORT-induced by defending against ROS generation and regulation of antioxidant pathway (Subermaniam et al., 2020). Jia et al. had evaluated the protective effect of *Cyperi Rhizoma* on corticosterone-induced PC12 cells by the regulation of cell sphingolipids metabolism and activation of endoplasmic reticulum stress (ERS) (Jia et al., 2019). In addition, BDNF is a crucial brain signaling protein associated with depression disorder that plays a vital role in neuronal plasticity. Lower expression of BDNF have been present in the prefrontal cortex and hippocampus of post-mortem brain from subjects with major depressive disorder (MDD) without antidepressant treatment, and antidepressant could restore normal BDNF levels (Chen et al., 2001; Nunes et al., 2018).

This study first used behavioral despair animal model to screen the antidepressant effects of ZHD-WE and ZHD-EE. Furthermore, we focused on exploring the neuroprotective effects of ZHD-EE on the corticosterone-induced apoptosis of PC12 cells and discussed whether the resulting neuroprotection was due to upregulation of the expression of the BDNF and restoring the balance of the Bcl-2/Bax apoptotic pathway.

## Materials and Methods

### Drugs and Reagents

Corticosterone was obtained from Tokyo Chemical Industry (TCI) Development Co., Ltd. (Shanghai); Fetal bovine serum (FBS) was obtained from Biological Industries; LDH Assay kit was purchased from Nanjing Jiancheng Bioengineering Institute; Annexin V-FITC/PI kit was purchased from Jiangsu Kaiji Biotechnology Co., Ltd. and MTT was obtained from Sigma-Aldrich.

Fluoxetine hydrochloride capsules (batch number: 5548C) were purchased from Lilly Suzhou Pharmaceutical Co., Ltd; Genipin 1-gentiobiside (batch number: B20637, purity ≥98%) was obtained from Shanghai Yuanye Biotechnology Co., Ltd. Geniposide (batch number: MUST-17020401, purity ≥98%), naringin (batch number: MUST-17040102, purity ≥98%), hesperidin (batch number: MUST-17032502, purity ≥98%), neohesperidin (batch number: MUST-17040707, purity ≥98%), honokiol (batch number: MUST-17020205, purity ≥98%) and magnolol (batch number: MUST- 18032102, purity ≥98%) were purchased from Chengdu Munster Biotechnology Co., Ltd. Methanol and acetonitrile are purchased from Merck. The ultrapure water for was prepared by a Milli-Q purification instrument. All other chemical reagents are of analytical grade.

### Preparation of ZHD-WE and ZHD-EE

Zhi-Zi Hou-Po Decoction is composed of *Gardenia jasminoides* Ellis (batch number: 20150301, origin: Hubei), *Citrus aurantium* L. (batch number: 150401, origin: Sichuan) and *Magnolia officinalis bark* (batch number: 20150301, origin: Sichuan). Three kinds of Chinese herbal medicine were obtained from Beijing Tongrentang Pharmacy and were authenticated by Qian Zhang from the Department of Pharmacy, Nanjing University of Chinese Medicine.

ZHD-EE samples were prepared according to a previous developed method in our laboratory (Bai et al., 2021). And the ZHD-WE was prepared by the water-boiling method. All crude herbs power was immersed in 75% ethanol or purified water (1:10, w/v) for 2 h, followed by reflux extraction or water-boiling method for 1 h. The extracted solution was filtered and the procedure was repeated again in a total volume of 8 times (1:8, w/v) weight of the herbs. The final filtrate concentration of two extracts of ZHD was 1.2 g ml^−1^. All the samples were stored at −20°C in the freezer prior to use.

### The Determination of ZHD Compounds

The method for the determination of seven compounds (genipin 1-gentiobioside, geniposide, naringin, hesperidin, neohesperidin, honokiol, and magnolol) in ZHD-WE and ZHD-EE was described in the light of the method previously developed by our laboratory (Bai et al., 2021). The results shown that the baseline separation of these ZHD-WE and ZHD-EE was achieved under optimum conditions (Supplementary Figure S1). The main difference between ZHD-WE and ZHD-EE is the content of honokiol and magnolol. The levels of honokiol and magnolol in ZHD-EE is higher than in ZHD-WE (honokiol:1.49 mg/g vs. 0.66 mg/g, and magnolol: 4.44 mg/g vs. 1.30 mg/g). These detailed contents of the seven components in ZHD-WE and ZHD-EE are presented in the Supplementary Table S1.

### Animals and Drug Administration

Eighty male ICR mice weighing 18–22 g were provided by the Animal Experimental Center of Nantong University. The mice were fed in the animal room with a fixed light/dark cycle (8:00–20:00), a room temperature of 23 ± 2°C, humidity of 40%–60% and free access to food and water (10 mice in each cage). The experiment was conducted according to the experimental design, and the whole experiment process was in accordance with internationally recognized principles for the use and care of laboratory animals. After adaptation for 7 days, the mice were divided into eight groups (*n = 10*), including the control group (normal saline), 4, 8, and 16 g kg^−1^ ZHD-WE groups, 4, 8, and 16 g kg^−1^ ZHD-EE groups, and a fluoxetine group (Flu, 20 mg kg^−1^). The mice were treated by gavage for seven consecutive days, and then TST and FST behavior tests were carried out after 1 h of final administration.

### Tail Suspension Test

A tail suspension test was performed according to a previously described method (Steru et al., 1985). Tested mouse was hung for 6 min with adhesive tape above the floor (approximately 1 cm from the tip of the tail). The tail suspension test was measured using the total duration of immobility after the first 2 min adaptation. Mice were considered immobile when they ceased struggling and they moved only to breathe. The last 4-min of immobility time was recorded by observers who were blind to the mice groupings.

### Forced Swimming Test

FST was performed according to a previously reported method (Porsolt et al., 1977). Tested mouse was individually kept in a transparent glass container (diameter, 14 cm and height 20 cm), containing water with a height of 12 cm. Mice were forced to swim for 6 min and the immobility time was recorded during the last 4 min. The mice were judged to be immobile when they ceased struggling and remained floating motionless, making movements only to keep their heads above water.

### Determination of Hippocampal BDNF Content

The mice were sacrificed by cervical dislocation, and then bilateral hippocampi were rapidly peeled off in ice dish. These hippocampal samples were precisely weighed, and cold physiological saline was added according to weight (g): volume (ml) at a ratio of 1:20. These samples were then centrifuged at 12,000 rpm for 10 min after homogenization. The BDNF were then measured according to the ELISA operating instructions (CUSABIO, Wuhan, China).

### Cell Culture

PC12 cells were donated by the central laboratory of Jiangsu Province Hospital of Chinese Medicine and maintained in DMEM (Thermo Fisher (Suzhou) Instrument Co., Ltd., Suzhou, China) supplemented with 10% of fetal bovine serum (Biological Industries Israel Beit Haemek Ltd, Kibbutz Beit Haemek, Israel), penicillin (100 unit/ml), streptomycin (100 μg/ml) in a water-saturated atmosphere at 5% CO_2_ and 37°C. The medium was changed every other day. PC12 cells were passaged by 0.25% trypsinization every two to 3 days and the cells within 30 generations were selected for follow-up experiments.

### PC12 Cell Viability

PC12 cells in exponential growth phase were seeded into 96-well plates (1 × 10^4^ cells/well) and left for 48 h prior to being cultured in different doses of ZHD-EE. To study the effect of ZHD-EE on the viability of PC12 cells, the cells were divided into nine groups: a control group (serum-free medium), 6.25, 12.5, 25, 50, 100, 200, 400, and 800 μg ml^−1^ ZHD-EE groups. Experiments were performed after 24 h incubation with serum-free medium and various doses of ZHD-EE. An aliquot (20 µl) of 5 mg/ml MTT solution was added to the well and PC12 cells were then incubated for 4 h at 37°C. The cultured supernatant was discarded, and 150 µl of dimethyl sulfoxide solution was added to each well. The solution was mixed in the dark at low speed for 10 min at room temperature to thoroughly dissolve the resulting blue-purple crystals. The optical density (OD) of each well was determined at 570 nm using a microplate reader. The cell survival rate (%) = (OD value of treatment group/OD value of control group) * 100%.

### Corticosterone Treatment

PC12 cells were seeded in 96-well plates for 48 h incubation (1 × 10^4^/well), and then treated with different concentrations of corticosterone (0, 50, 100, 200, 400, and 800 μM) for another 24 h. The cells were then harvested and the OD value were detected by the MTT method. The optimal CORT concentration was chosen when the cell viability decreased to approximately 50% for subsequent experiments (Jiang et al., 2015).

### Neuroprotective Effects of ZHD-EE on CORT-Treated PC12 Cells

In subsequent experiments, exponentially growing PC12 cells were seeded in 96-well plate, and then different doses of ZHD-EE and 400 μM corticosterone were added. The PC12 cells were divided into eight groups: a control group, 6.25 μg ml^−1^ ZHD-EE group, 12.5 μg ml^−1^ ZHD-EE group, 25 μg ml^−1^ ZHD-EE group, 50 μg ml^−1^ ZHD-EE group, 100 μg ml^−1^ ZHD-EE group, 200 μg ml^−1^ ZHD-EE group, and 400 g ml^−1^ ZHD-EE group. The control group was cultured with basic medium. MTT assay results were measured after 24 h of co-incubation with corticosterone and different concentrations of ZHD-EE.

### Lactate Dehydrogenase Release Assay

PC12 cells were cultured at a density of 1 × 10^4^ cells per well in 96-well microplates and incubated for 48 h. And then PC12 cells were treated with 400 μM corticosterone and low, middle and high doses ZHD-EE (25, 100, and 400 μg ml^−1^, respectively). The control group was cultured in basic medium. LDH measurement was conducted using cells supernatants and an LDH assay kit (Nanjing Jiancheng Bioengineering Institute) according to the manufacturer’s protocol. In brief, pyruvate, matrix buffer and coenzyme I solution were added in turn to the cell supernatants and incubated at 37°C for 15 min. Next, 2,4-dinitrophenylhydrazine was put into and incubated for 15 min. Finally, 0.4 mol/L NaOH solution was used to stop the reaction. The OD values were then determined at 450 nm.

### Apoptosis Detection by Flow Cytometry

Cell apoptosis was measured in accordance with the instructions of an Annexin-V-FITC/PI apoptosis kit. Briefly, harvested cells were collected at the end of treatment and washed twice with PBS. Next, the PC12 cells were re-suspended in 300 μl of binding buffer. Following this, the cells were incubated with FITC-labelled Annexin V (5 μl) and PI (10 μl) at room temperature for 15 min. PC12 cell apoptosis rate was determined using a FACS Calibur flow cytometer.

### Reverse Transcription-Quantitative PCR

Expression of Bcl-2, Bax and BDNF mRNA were examined by real-time RT-PCR and glyceraldehyde-3-phosphate dehydrogenase (GAPDH) gene was used as an internal reference. PC12 cells were seeded in a 6-well plate (1 × 10^6^ cells/well) and harvested after treatment with different doses of ZHD-EE for 48 h with or without 400 μM corticosterone and total RNA was extracted using TRIzol reagent, following the instructions provided by the manufacturer. RNA purity was considered acceptable for ratios of the absorbances at 260 and 280 nm between 1.8 and 2.1 using a UV spectrophotometer. RT-qPCR reactions were performed on an ABI StepOnePlus Real-Time PCR System with 40 cycles 95°C for 15 s, 60°C for 20 s, and 72°C for 40 s. The sequences of were as follows: BDNF forward: 5-ATT​AGC​GAG​TGG​GTC​ACA​GC-3′, reverse: 5′-GTAGTTCGGCAT TGCGAGTT-3′; rat Bax forward: 5-CCA​GGA​CGC​ATC​CAC​CAA​GAA​G -3′, reverse: 5-GCCACA CGG​AAG​AAG​ACC​TCT​C-3′; rat Bcl-2 forward: 5′-CCT​GGT​GGA​CAA​CAT​CGC​TCT​G-3′, reverse: 5-GCA​TCC​CAG​CCT​CCG​TTA​TCC​T-3′; rat GAPDH forward: 5-AGGTTGTCTCCT GTGACTTCAA-3′, reverse: 5-CTG​TTG​CTG​TAG​CCA​TAT​TCA​TTG-3′. The relative levels of Bcl-2, Bax and BDNF were calculated using the 2^−ΔΔCt^ method.

### Western Blot Analysis

PC12 cells were seeded in 6-well plates and incubated with different doses of ZHD-EE for 48 h with or without 400 μmol L^−1^ corticosterone. Harvested cells were washed with PBS and then lysed with 200 μl RIPA lysis buffer containing 1% phenylmethylsulfonyl fluoride and cocktail protease inhibitors for 0.5 h on ice. The amounts of proteins were measured according to the instructions of BCA protein detection kit (Beyotime Biotechnology, Jiangsu, China). Proteins were separated on 12% SDS-PAGE gels and then transferred to PVDF membrane by wet method. The membrane was soaked in PBST with 5% non-fat milk and incubated with antibodies against Bcl-2, Bax, BDNF and β-actin overnight at 4°C, followed by additional incubation with secondary antibody for 1 h. The protein bands were quantified using a chemiluminescent imaging system and the protein expression was normalized to β-actin.

### Statistical Analyses

All results are expressed as the mean ± SD (standard deviation). Figures were prepared using GraphPad Prism 6.0 software (San Diego, CA, United States) and SPSS 18.0 (IBM, Chicago, IL, United States) was used for testing the differences between multiple groups by ANOVA followed by Dunnett’s post hoc test. A value of *p* < 0.05 was considered statistically significant.

## Results

### Effects of ZHD-WE and ZHD-EE on Immobility Time in TST and FST

**FIGURE 1:**
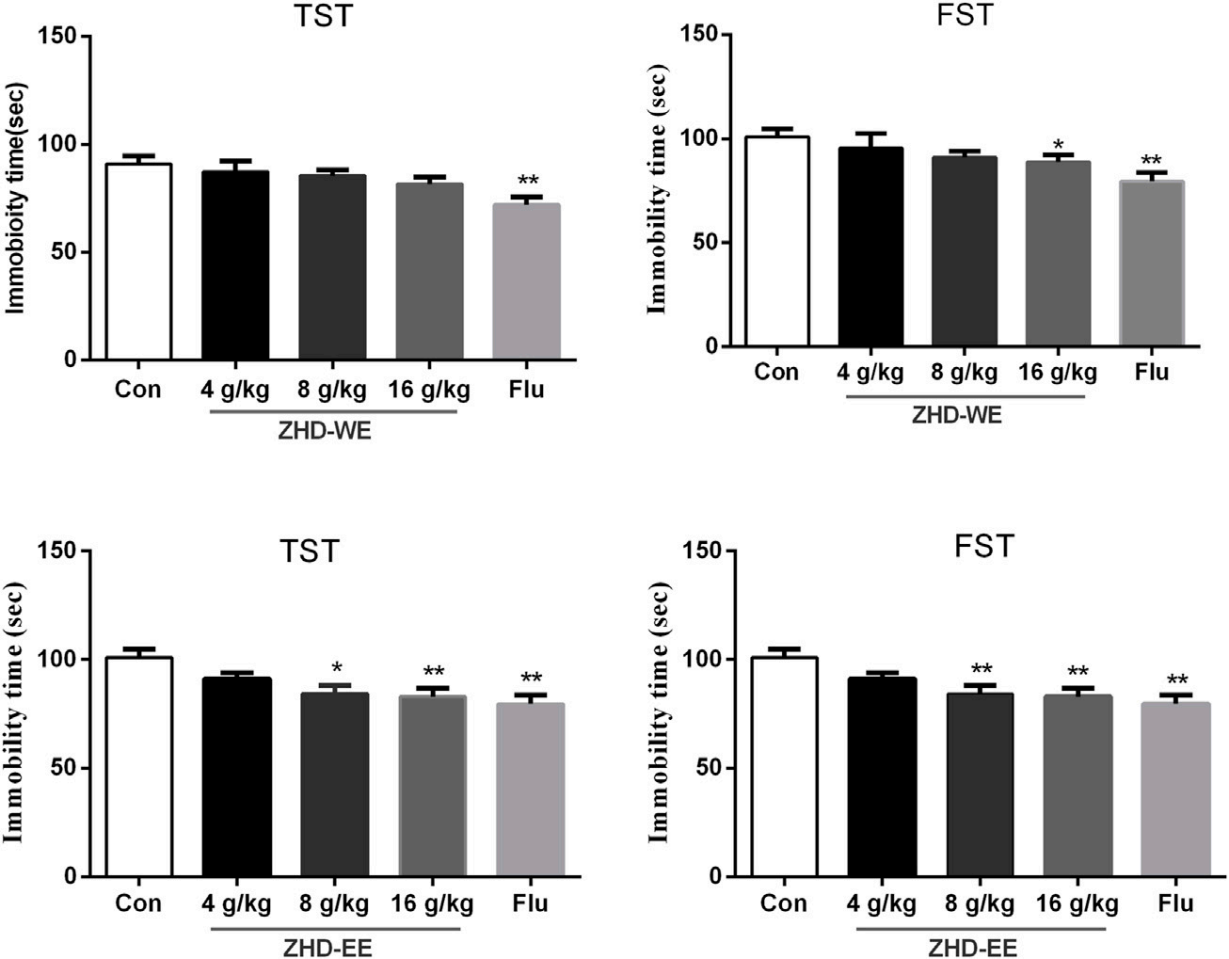
Effects of ZHD-WE and ZHD-EE on the immobility time in tail suspension test and forced swimming test. The data are presented as the mean ± SD (*n =* 10). ZHD-WE, water extract of ZHD; ZHD-EE, ethanol extract of ZHD.

As shown in Figure 1, treatment with fluoxetine significantly shortened the immobility time in TST and FST compared with the control group (*p* < 0.01, *p* < 0.01, respectively). After the mice were orally administered ZHD-EE for seven consecutive days, compared to the control group, treatment with 16 g kg^−1^ dose of ZHD-WE shortened the immobility time in the FST (*p <* 0.05), and other doses had no effect on immobility time in the FST and TST. In addition, the results showed that 4 g kg^−1^ doses of ZHD-EE did not show an obvious effect in FST and TST, while 8 and 16 g kg^−1^ doses of ZHD-EE group led to a marked decrease in immobility duration in comparison with the control group in both FST and TST (Figure 1).

### The Levels of BDNF in *Hippocampus*

**FIGURE 2:**
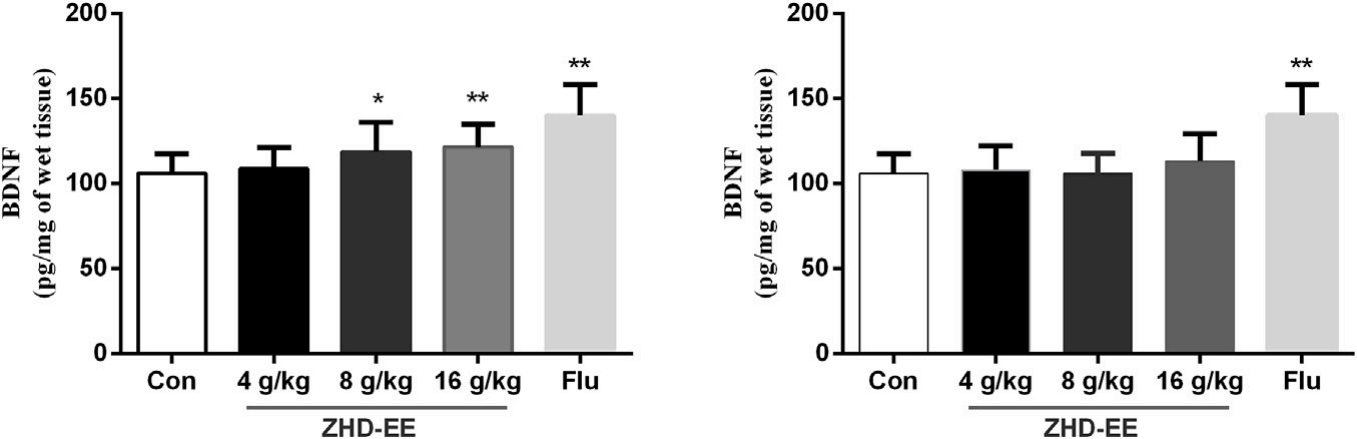
Effects of ZHD-EE and ZHD‐WE on the BDNF levels in the hippocampus. The data are presented as the mean ± SD (*n* = 10). ZHD‐EE: ethanol extract of ZHD; ZHD‐WE: water extract of ZHD.

In order to further study the potential mechanism of antidepressant effect of ZHD, we measured the level of BDNF using ELISA in the hippocampus. As shown in Figure 2, the mice administered with the doses of ZHD-EE (8 and 16 g kg^−1^) could effectively increase BDNF expression of hippocampus in comparison with the control group (*p* < 0.05 or *p* < 0.01, respectively). However, the BDNF level could not be regulated by all of these doses of ZHD-WE (*p* > 0.05). As expected, fluoxetine significantly increased BDNF expression in the hippocampus (*p* < 0.01). It supports that BDNF plays an important role in the antidepressant effect of the ZHD-EE.

### ZHD-EE Effects on the Viability of PC12 Cells

**FIGURE 3:**
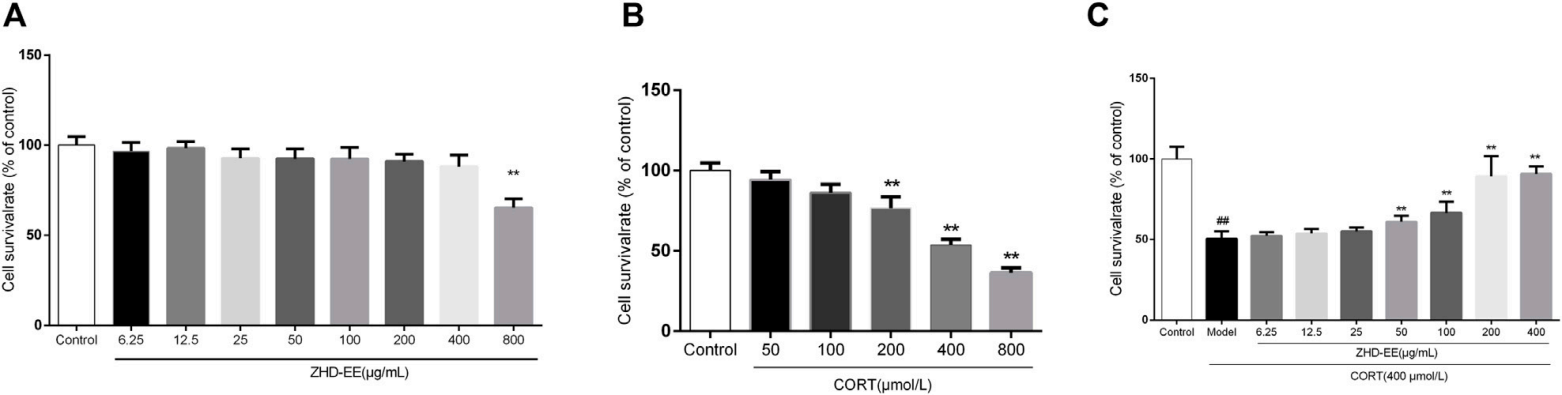
The cell viability was measured by MTT assay. **(A)**: PC12 cells were exposed to different concentrations of ethanol extract of ZHD-EE (6.25, 12.5, 25, 50, 100, 200, 400, and 800 μg·mL^‒1^); **(B)**: PC12 cells were exposed to different concentrations of CORT (50, 100, 200, 400, and 800 μM) for 24 h; **(C)**: PC12 cells were exposed to different concentrations of ethanol extract of ZHD-EE (6.25, 12.5, 25, 50, 100, 200, and 400 μg·mL^‒1^) and 400 μM CORT for 24 h. *^##^P* < 0.01 vs. control group, **P < 0.01 vs. model group. ZHD-EE: ethanol extract of ZHD.

As shown in Figure 3A, results from treatment with ZHD-EE at doses of 6.25, 12.5, 25, 50, 100, 200, 400, 800 μg ml^−1^ for 24 h showed that 800 μg ml^−1^ ZHD-EE markedly decreased cell viability. In addition, the cell survival rate decreased after exposure to 200–800 μM corticosterone. Compared with the control group, the PC12 cells co-incubated with corticosterone at doses of 200, 400, and 800 μM for 24 h had survival rates that decreased to 76.7, 53.6, and 36.3%, respectively (Figure 3B). The obtained data showed that 400 μM corticosterone was an appropriate damage concentration. Compared with the control group, PC12 cells survival rate in the model group decreased significantly, and the ZHD-EE at doses of 50 (*p* < 0.01), 100 (*p < 0.01*), 200 (*p < 0.01*), and 400 μg ml^−1^ (*p* < 0.01) could reverse the decrease in cell survival rate induced by corticosterone (Figure 3C).

### LDH Activity

**FIGURE 4:**
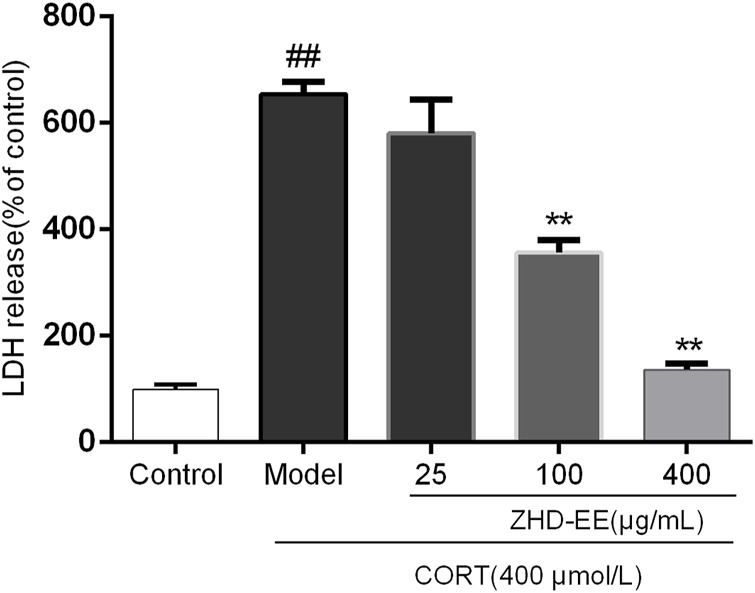
Effects of ZHD-EE (25, 100, and 400 μg·mL^−1^) on the LDH leakage in CORT-treated PC12 cells. The values given are the mean ± SD (*n =* 3); ^##^ *p <* 0.01 compared with the control group; **p < 0.01 compared with the model group. ZHD-EE: ethanol extract of ZHD.

As can be seen from Figure 4, the LDH release rate in the model group was significantly higher than that in the normal group. However, 100 and 400 μg ml^−1^ ZHD-EE could markedly reduce the LDH release from PC12 cells (*p <* 0.01 or *p <* 0.01, respectively).

### PC12 Cell Apoptosis

**FIGURE 5:**
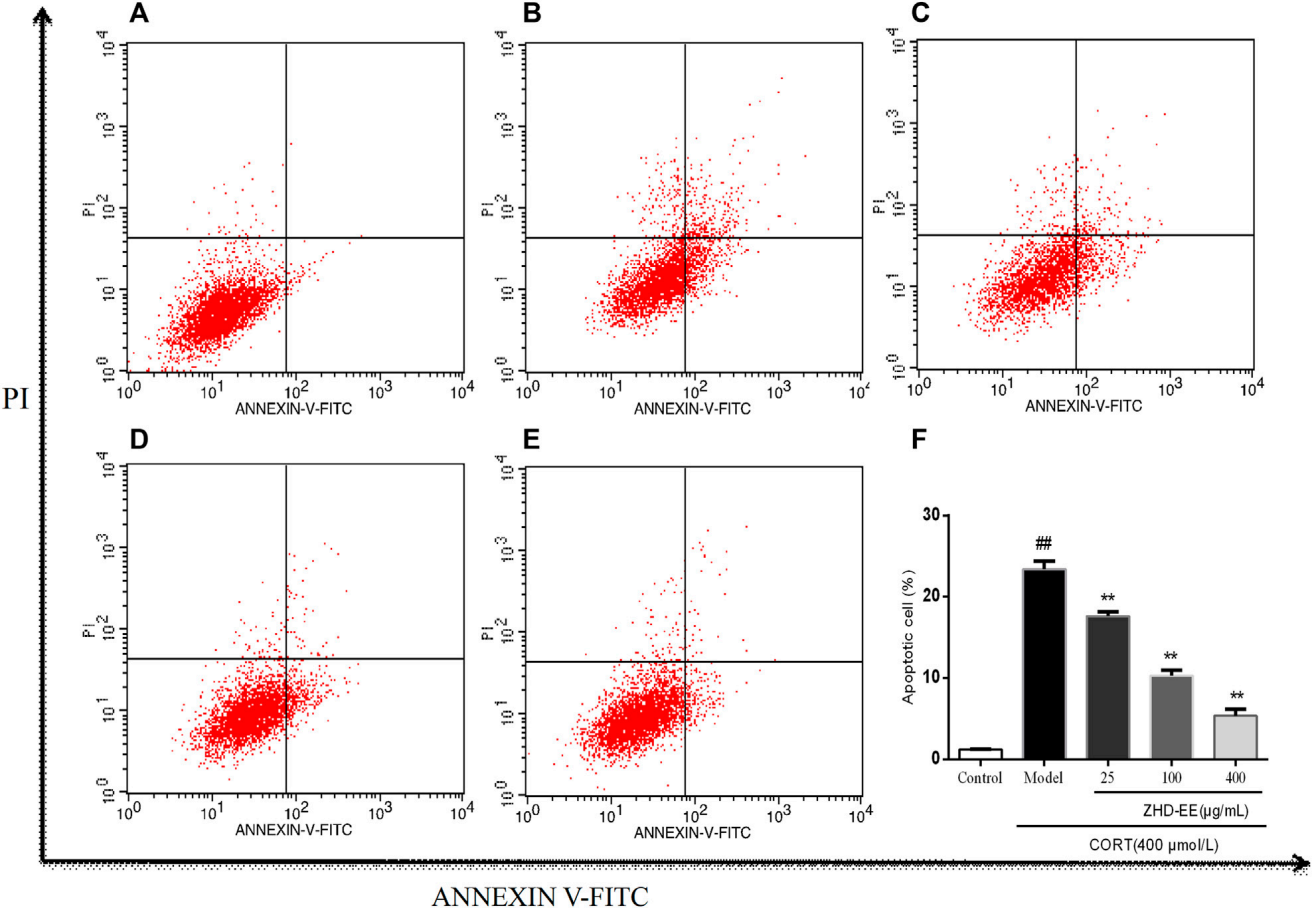
Effects of ZHD-EE on the cell apoptotic rates in CORT-treated PC12 cells by Annexin V-FITC/PI double staining with flow cytometry. **(A)**: Normal cell; **(B)**: PC12 cell lesioned by 400 μM CORT; **(C−E)**: PC12 cell treated by 25, 100, 400 μg·mL^−1^ ZHD-EE and 400 μM CORT. **(F)**: the histogram of cell apoptosis rate. ^##^ *p <* 0.01 compared with the control group, ^**^ *p <* 0.01 compared with the CORT-treated group. ZHD-EE: ethanol extract of ZHD.

Annexin V-FITC and PI double staining were used to determine the effect of ZHD-EE on PC12 cells injured by corticosterone. As shown in Figure 5, abundant apoptotic cells were produced in the model group, after treatment with 400 μM corticosterone for 24 h, the cells apoptosis rate increased from 1.2% in the control group to 23.4%. On the contrary, apoptotic cells was overtly improved after treated with ZHD-EE. The total apoptosis rate of PC12 cells significantly decreased when co-incubated with 25, 100, or 400 μg ml^−1^ ZHD-EE (*p* < 0.01, *p* < 0.01, *p* < 0.01, respectively), demonstrating that ZHD-EE had a protective effect against apoptosis in PC12 injury cells.

### BDNF, Bcl-2, and Bax mRNA and Proteins Levels

**FIGURE 6:**
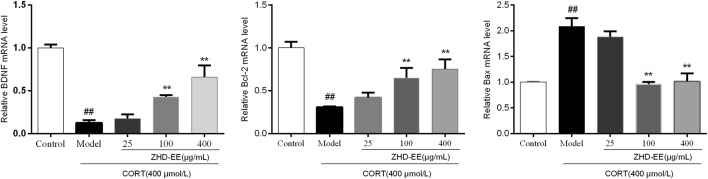
Effect of ZHD-EE on the BDNF, Bcl-2 and Bax mRNA in CORT-treated PC12 cells. Data are expressed as a percentage of the control and the results are expressed as the mean ± SD (*n =* 3). ^##^ *p <* 0.01 compared with the control group, **p < 0.01 compared with the CORT-treated group. ZHD-EE: ethanol extract of ZHD.

**FIGURE 7:**
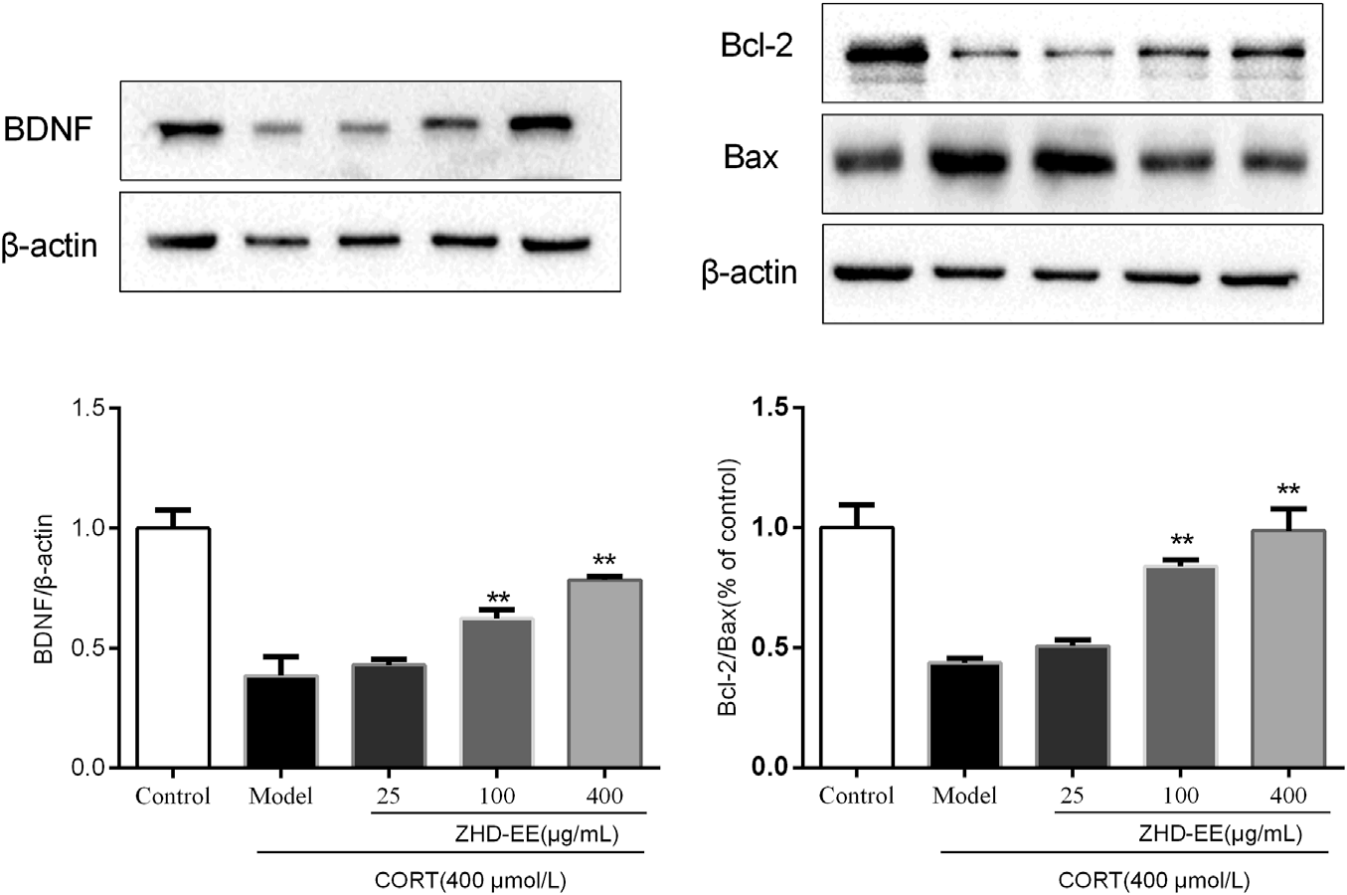
Effects of ZHD-EE on the protein expression of BDNF, Bcl-2 and Bax in corticosterone-treated PC12 cells by western blot analysis. The data are presented as the mean ± SD (*n =* 3). ^##^ *p <* 0.01 compared with the control group, **p < 0.01 compared with the CORT-treated group. ZHD-EE: ethanol extract of ZHD.

BDNF is a crucial brain signaling protein associated with depression disorder that plays a vital role in neuronal plasticity. In addition, studies have shown that hippocampal cell apoptosis is involved in the pathological changes of hippocampus in patients with depression. As shown in Figure 6 and Figure 7, compared with the control group, the levels of BDNF and Bcl-2 mRNA and proteins in the CORT model group significantly decreased, and the Bax mRNA expression significantly increased (*p* < 0.01, *p* < 0.01, *p* < 0.01, respectively). Meanwhile, the results showed that 100 and 400 μg ml^−1^ ZHD-EE treatment could reverse the expression of the abnormal expression of Bax, Bcl-2 and BDNF. The obtained data showed that ZHD-EE had a protective effect on CORT-induced injury in PC12 cells by upregulating the expression of BDNF and restoring Bcl-2/Bax balance.

## Discussion

TCM has received much attention over the past decades in psychopharmacology because of the various adverse events in western medication. Mounting research has demonstrated that the herbal mechanisms used for depression involve hypothalamic-pituitary-adrenal axis, re-uptake of neurotransmitter, neuroprotection, neural plasticity, and so on (Liu et al., 2015). Development of antidepressants requires behavioral tests for preliminary screening before undertaking more complex preclinical tests (Castagne et al., 2011). A forced swimming test and a tail suspension test in mice are simple to operate and have high specificity for evaluating the efficacy of antidepressants. The obtained data from TST and FST showed that the ethanol extract of ZHD at concentrations of 8 and 16 g kg^−1^ significantly decreased the immobility time, and only high concentration of water extract of ZHD could significantly decreased the immobility time in the FST. ZHD-EE showed better antidepressant effect compared with ZHD-WE in this study. Previous studies found that water extract of ZHD has antidepressant effect (Xing et al., 2015; Xing et al., 2019). We think that the inconsistent results may be caused by the following reasons: This study used the despair model (TST and FST) to screen the antidepressant effect of ZHD according to the compatibility proportion of Gardenia jasminoides Ellis fruit, Citrus aurantium L. fruit and Magnolia officinalis Rehd. et Wils. Bark (1:1:1.2) according to previous report (Meng et al., 1998). While Xing et al. explored the antidepressant effects of ZHD (water extract) in a rat model of chronic unpredictable mild stress at the compatibility proportion of Gardenia jasminoides Ellis fruit, Citrus aurantium L. fruit and Magnolia officinalis Rehd. et Wils. Bark (1:1:7). The dosage of Magnolia officinalis was different in these studies. Modern pharmacological studies have demonstrated that the active compounds of lignans, iridoids and flavonoids in ZHD exert antidepressant effects. Among the compounds in ZHD, magnolol and honokiol are considered to be the major antidepressant ingredients of ZHD (Xu et al., 2008; Qiang et al., 2009). Moreover, pharmacodynamics studies have shown that magnolol and honokiol could enter the brain through the blood brain barrier (Liu et al., 2016; Zhu et al., 2020). The dosage of Magnolia officinalis in Xing et al.’ study is larger and therefore the content of magnolol and honokiol in ZHD (water extract) is higher than that our ZHD (water extract), which maybe lead to poor antidepressant effect in our water extract of ZHD. While the levels of magnolol and honokiol are higher in our ZHD (ethanol extract) because of strong lipid solubility of these ingredients. Therefore, in our study, ZHD-EE showed good antidepressant effect compared with ZHD-WE.

It has been reported that stress leads to the hyperactivity of the HPA axis, which participates in stress response and causes an increase in the corticosterone level under the action of adreno-cortico-tropic-hormone (ACTH). Excessive corticosterone level can damage normal hippocampal neurons and eventually lead to depression (Murray et al., 2008). Increasing evidence indicates that a certain concentration of corticosterone damages PC12 cells, and antidepressants could effectively protect these cells from CORT-induced cytotoxicity. In our study, PC12 cells injury model was used to study the protective effects of ZHD. The results of MTT and LDH assays showed that the survival rate of PC12 cells decreased with an increase in corticosterone concentration. However, the cell survival rate significantly increased after pretreatment with 50, 100, 200, or 400 μg ml^−1^ ZHD-EE. Meanwhile, ZHD-EE at concentrations of 100 or 400 μg ml^−1^ could significantly reduce the release rate of LDH, which confirmed that ZHD-EE had neuroprotective effect in PC12 cells injured by corticosterone. In addition, the apoptosis of PC12 cells was determined using Annexin V-FITC/PI double staining, and the results showed that corticosterone induced obvious apoptosis in PC12 cells, while 25, 100, or 400 μg ml^−1^ of ZHD-EE could effectively reverse this apoptosis in a dose-dependent manner. In our study, the dose administered to the mice was based on the clinical dose. The dose administered to the mice is inconsistent with the consensus document in the literature (Heinrich et al., 2020). However, before we did the experiment, we consulted some literature on dosage of traditional Chinese medicine extracts for animals, and found some animal doses in experiments were also relatively high (Gong et al., 2019; Xing et al., 2019; Li et al., 2021).

Previous studies had confirmed that apoptosis of hippocampal cells in rat increase in CUMS, and antidepressant treatment significantly reduce the number of apoptotic cells, which indicated that apoptosis was involved in the pathological changes of the hippocampus in depression (Lucassen et al., 2004). Apoptosis is one of the main mechanisms of neuronal injury, in which the Bcl-2 family of proteins plays an important role in regulating neuronal survival (Antony and Vijayan, 2016). Bcl-2 and Bax, as one of the last pathways of apoptosis, are closely related to depression. The results showed that the expression of Bax markedly increased, while the expression of Bcl-2 significantly decreased in PC12 cells treated with corticosterone. Pretreatment with ZHD-EE could significantly downregulate the expression of Bax, and upregulate the expression of Bcl-2, suggesting that ZHD-EE can change the expression of apoptosis-related mRNA and proteins, and inhibit corticosterone-induced apoptosis in PC12 cells.

The BDNF hypothesis has highlighted the important role of plasticity in depression, as this is an important member of the neurotrophin family that participates in the growth of nerve cells and regulates synaptic plasticity and physiological function (Xu et al., 2020). Clinical studies have confirmed that BDNF levels in plasma are decreased in case of depression (Monteleone et al., 2008), and antidepressant treatment can increase BDNF levels (Polyakova et al., 2015). In our study, animal experiments showed that the BDNF level was significantly improved after administration with ZHD-EE for seven consecutive days, and the results *in vitro* showed the expression of BDNF mRNA and protein decreased significantly in PC12 cells subjected to CORT, while pretreatment with 100 or 400 μg mL^−1^ doses of ZHD-EE could significantly increase the expression of BDNF mRNA and protein.

## Conclusion

The obtained findings in our study revealed that the ethanol extract of ZHD had significantly improved despair-like behavior in mice and produced neuroprotective effects in PC12 cells injury model induced by corticosterone. Further studies showed that its mechanism may up-regulate the expression of BDNF and alter the ratio between Bcl-2 and Bax to inhibit PC12 cell apoptosis. This study confirmed that the ethanol extract of ZHD had neuroprotective effects at the cellular level, which provides a new experimental basis for the clinical practice of ZHD in depression.

## Data Availability

The original contributions presented in the study are included in the article/Supplementary Material, further inquiries can be directed to the corresponding authors.

## Abbreviations

ZHD, Zhi-Zi Hou-Po Decoction; ZHD-WE, water extract of ZHD; ZHD-EE, ethanol extract of ZHD; CORT, corticosterone; FST, forced swimming test; TST, tail suspension test; BDNF, brain-derived neurotrophic factor; LDH, lactate dehydrogenase; TCM, traditional Chinese medicine; MDD, major depressive disorder; RT-qPCR, Reverse Transcription-quantitative PCR.
