# Supplementary figures and images for: Potential Anti-Depressive Effects and Mechanisms of Zhi-Zi Hou-Po Decoction Using Behavioral Despair Tests Combined With in Vitro Approaches
Source: Front Pharmacol. 2022 Jul 6;13:918776. doi: 10.3389/fphar.2022.918776 (PMC9298739; doi:10.3389/fphar.2022.918776)

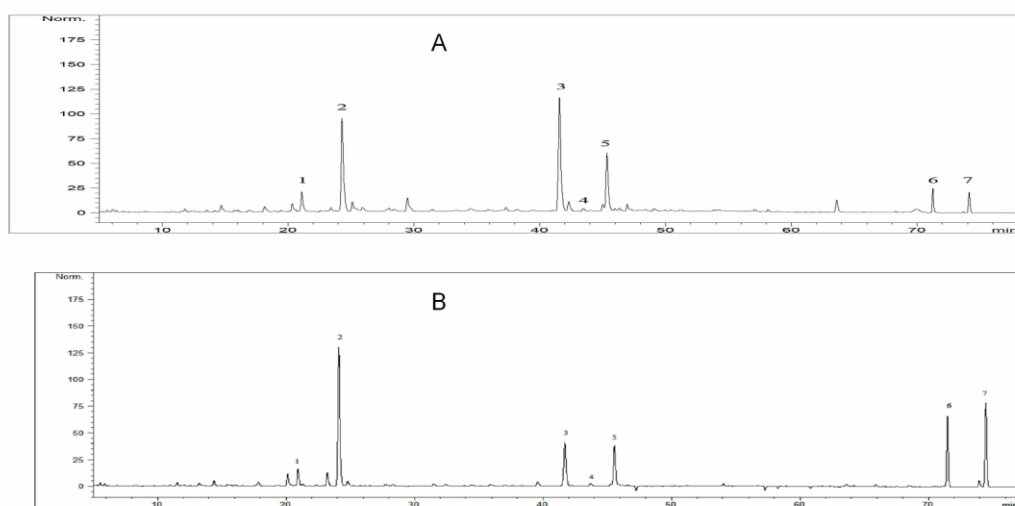

Figure S1 The chromatographic peak of the seven components in ZHD-WE (A) and ZHD-EE (B) are presented

Supplement: Supplementary file 1 [file DataSheet1.PDF]
